# Isolation and Characterization of Saponin-Producing Fungal Endophytes from *Aralia elata* in Northeast China

**DOI:** 10.3390/ijms131216255

**Published:** 2012-11-30

**Authors:** Hao Wu, Hongyan Yang, Xiangling You, Yuhua Li

**Affiliations:** College of Life Sciences/Daqing Bio-tech Research Institute, Northeast Forestry University, Harbin 150040, Heilongjiang, China; E-Mails: mad_knight@163.com (H.W.); cnyanghy@163.com (H.Y.); yxiangling@yahoo.com (X.Y.)

**Keywords:** endophytic fungi, saponin, *Aralia elata*, microbial diversity

## Abstract

The purpose of this study was to investigate the diversity of endophytic fungi of *Aralia elata* distributed in Northeast China as well as their capacity to produce saponins. Ninety-six strains of endophytic fungi were isolated, and polymerase chain reaction (PCR) and sequencing were employed to identify the isolates. The saponin concentrations of the culture filtrates of representative strains were measured. The agar diffusion method was used to test antimicrobial activity, while high-performance liquid chromatography (HPLC) was employed to identify the saponins produced by representative strains. *Alternaria*, *Botryosphaeria*, *Camarosporium*, *Cryptosporiopsis*, *Diaporthe*, *Dictyochaeta*, *Penicillium*, *Fusarium*, *Nectria*, *Peniophora*, *Schizophyllum*, *Cladosporium* and *Trichoderma* species were isolated in this study. Overall, 25% of the isolates belonged to *Diaporthe* (*Diaporthe eres*), and 12.5% belonged to *Alternaria*. The highest concentration of saponins was produced by G22 (2.049 mg/mL). According to the results of the phylogenetic analysis, G22 belonged to the genus *Penicillium*. The culture filtrate of G22 exhibited antibacterial activity against *Staphylococcus aureus*, and ginsenosides Re and Rb2 were detected in G22 culture filtrates by HPLC.

## 1. Introduction

Fungi that colonize the living internal tissues of plants without causing any immediate and overt negative effects, have been called endophytic fungi [1]. Endophytic fungi have been found to be ubiquitous within all types of plants [2–4], and to play an important role in the ecological community. Some endophytic fungi have been found to influence their host’s growth [5], enhance stress resistance [6], degrade pollutants [7], and produce biologically active substances [8].

Some biologically active substances produced by plants, can also be derived from endophytes of host plants. The most famous substance of this class is taxol, a mitotic inhibitor used in cancer chemotherapy. This compound was originally produced by the yew tree, but it can also be produced by their endophytic fungi [9]. In addition, endophytic fungi isolated from *A. indica* can secrete azadirachtin A and B, which are used to repel insects [10].

*Aralia elata*, which is native to Asia, is a medicinal plant belonging to the family *Araliaceae*[11]. *A. elata* can produce saponins [12]. Saponins produced by medical plants have multiple therapeutic values, including being protective against cancer [13,14] and acting as a therapeutic agent for hepatitis and gastric ulcer [15,16]. Endophytic fungi in *A. elata* have not been well studied. Previously, Paul *et al.* reported the presence of endophytic fungi in roots of *A. elata* cultivated in Korea from the Chungnam province. In their study, the authors tested the antifungal activity against plant pathogenic fungi. Twenty-four genera were characterized, and *Strumella*, *Rhizopycnis* and *Entrophospora* were the most abundant taxa. Four isolates of *Pyrenochaeta*, 1 isolated of *Entrophospora* and 1 unidenitified species were positive against 6 plant pathogenic fungi tested [11]. To the best of our knowledge, there are no reports on endophytic fungi isolated from wild *A. elata* grown in Northeast China. In the present study, we investigated the diversity of the endophytic fungi harbored in populations of *A. elata* widely distributed in the Xiaoxing’anling area (Heilongjiang, China). The saponin production and antimicrobial activity of typical strains were analyzed.

## 2. Results and Discussion

### 2.1. Strains Identification and Phylogenetic Analysis

Ninety-six strains were isolated. Genomic DNA was extracted and the 28S rDNA D1/D2 region was amplified and sequenced. The obtained sequences were compared with those in the GenBank database, and the results are shown in Table 1.

**Figure 1:**
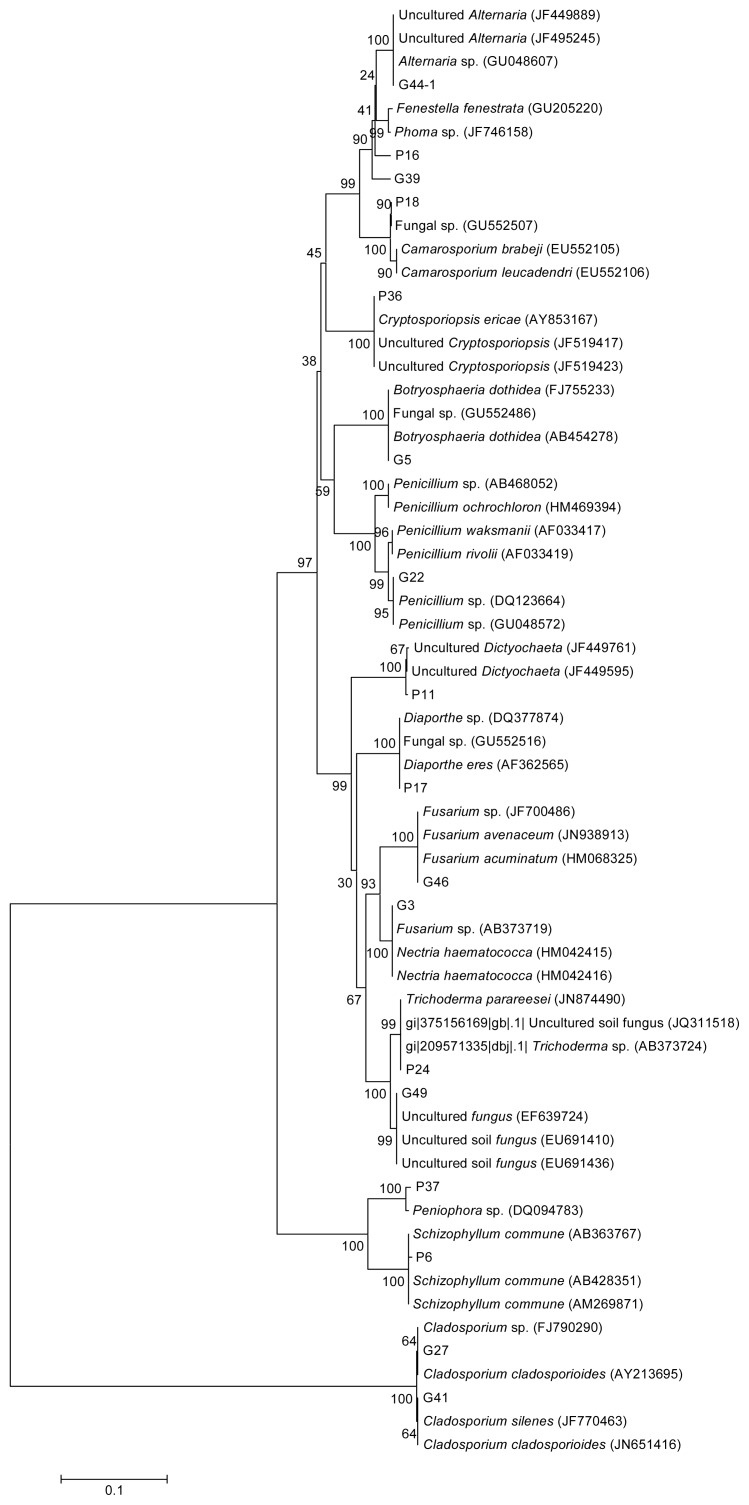
A 28S rDNA sequence-based phylogenetic tree constructed using the neighbor-joining method. Scale bar represents 10% estimated sequence divergence. Numbers in the branches indicate bootstrap values (percentages for 1000 replicates).

A phylogenetic tree built from the 28S rDNA sequences is shown in Figure 1. From Table 1 and Figure 1, the identified fungi included: *Alternaria*, *Botryosphaeria*, *Camarosporium*, *Cryptosporiopsis*, *Diaporthe*, *Dictyochaeta*, *Penicillium*, *Fusarium*, *Nectria*, *Peniophora*, *Schizophyllum*, *Cladosporium* and *Trichoderma*. The most abundant genera were *Diaporthe* and *Alternaria* with 25 and 12.5% of the total number of isolates, respectively. G49 was not identified because its sequence was significantly similar to unknown fungal sequences in the GenBank database.

*Diaporthe* and *Altenaria* were found to be the predominant genera, a finding that is different from previous studies showing that *Strumella*, *Rhizopycnis* and *Entrophospora* were the most abundant taxa in *A. elata*[11]. A possible reason for this discrepancy is that the *A. elata* specimens were derived from different regions [17].

*Diaporthe* are endophytic fungi that grow in several of plant species and have been shown to produced different secondary metabolites. For example *Diaporthe* sp. isolated from *Espeletia* sp. can inhibit the growth of *Phytophthora infestans*, a plant pathogen [18]. While *Diaporthe* sp. from *Curcuma longa* can convert curcumin into colorless hydroderivatives. Curcumin has a potent antioxidant effect. However, the distinct yellow color limits its use. This conversion may expand its application [19]. *Diaporthe* sp. P133, isolated from *Pandanus amaryllifolius*, can secrete benzopyranones, which inhibit a virulent strain of *Mycobacterium tuberculosis*[20]. *Diaporthe phaseolorum* isolated from mangrove forest can produce the antibacterial agent 3-hydroxypropionic acid [21]. A new varied species *Alternaria alternata* from the bark of 200-year-old *Taxus cuspidate* could produce taxoids of type III with the anti-neoplastic action [22]. *Alternaria* sp. isolated from *Brassica juncea* have demonstrated potential applications in biofuel feedback [23]. To the best of our knowledge, there are no reports concerning *Diaporthe* and *Altenaria* isolated from *A. elata*.

### 2.2. Analysis of Triterpenoid Saponins and Antimicrobial Activity

The concentration of triterpenoid saponin in the representative isolate from each group (Table 1) is shown in Table 2. The highest concentration of saponins was found in G22 (2.049 mg/mL), and this concentration is significantly higher than the level observed in P11 and P18 (*p* < 0.05). According to the results of phylogenetic analysis, G22 was identified as a *Penicillium* sp., P11 was identified as a *Dictyochaeta* sp., and P18 was identified as *Camarosporium* sp. The saponin concentrations among the strains of the same genus, such as G22 (2.049 mg/mL) and P23 (0.049 mg/mL), were significantly different (*p* < 0.05).

The growth-promoting factors and metabolites produced by endophytic fungi have been widely investigated and applied in both medicine and agriculture. The most notable substance produced by host endophytic fungi is taxol, a mitotic inhibitor used in cancer chemotherapy, and was originally produced by the yew tree [9]. Saponins produced by *A. elata* have multiple therapeutic values. The culture filtrates of endophytic fungi were analyzed to identify endophytes that produce triterpenoid saponins. It is well known that *Penicillium* is the source of penicillin, and recent studies show that endophytic *Penicillium* sp. also has the capacity to secrete anti-tumor substances [24,25] and gibberellin [26,27]. In this study, G22 (*Penicillium* sp.) has the high capacity to produce triterpenoid saponins.

From Table 3, G22 exhibited antibacterial activity against Gram-positive bacterium *Staphylococcus aureus* ACCC10499. P11 inhibited the growth of *S. aureus* ACCC10499, *Rhizoctonia solani* ACCC36233 and *Fusarium sporotrichioides*. At the same time, it expressed strong inhibition to *Klebsiella pneumoniae* ACCC10498. P18 also showed inhibition to *K. pneumoniae* ACCC10498.

### 2.3. Ginsenosides Analyses

**Figure 2:**
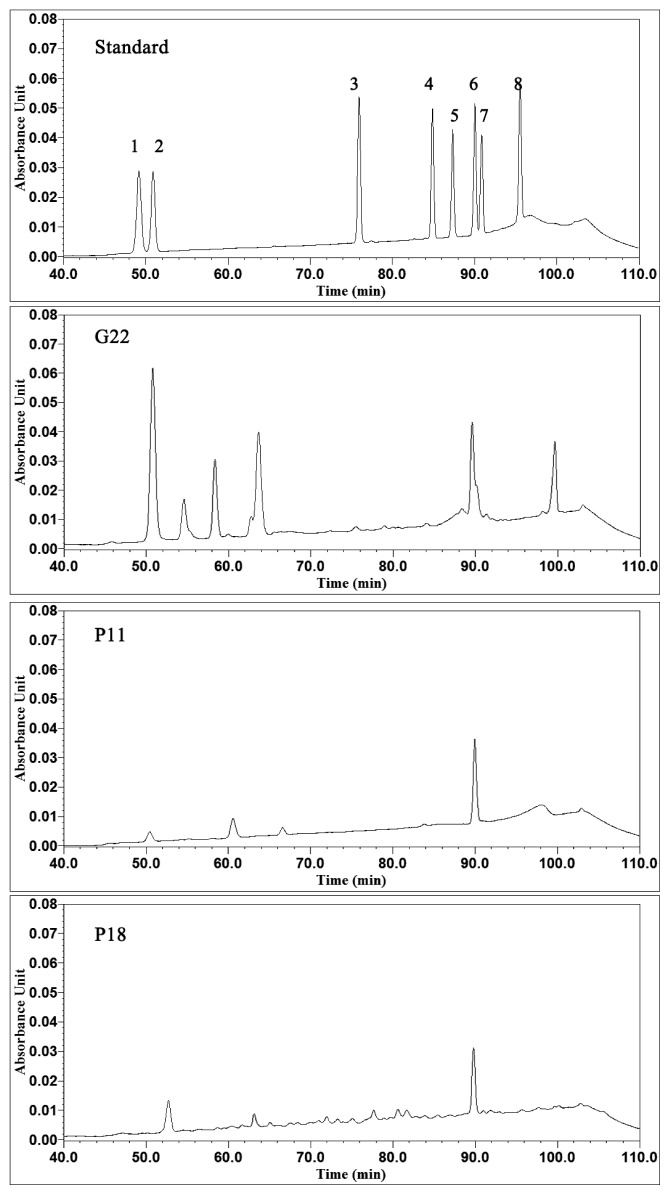
High-performance liquid chromatography (HPLC) spectra of the ginsenoside standards and the culture filtrates of the representative strains. 1, Rg1; 2, Re; 3, Rf; 4, Rb1; 5, Rc; 6, Rb2; 7, Rb3; 8, Rd.

To further analyze the composition of saponins, the culture filtrates of G22, P11, and P18, underwent HPLC. As a reference, eight ginsenoside standards were also analyzed. According to the spectra, which are shown in Figure 2, G22, P11 and P18 all produced detectable concentrations of saponins. Ginsenoside Rb2 was detected in the G22, P11, and P18 culture filtrates. More peaks were observed in the G22 culture filtrate. Ginsenoside Re was also detected in the G22 culture filtrate. These results indicate that the three strains have the capacity to produce ginsenosides, especially for G22. Additionally, G22 inhibited *S. aureus* growth (Table 3). These results indicate that G22 has a great potential for the further detailed study.

## 3. Experimental Section

### 3.1. Sampling and Isolation

Wild *A. elata* plants (5 years old) were sampled from the Xiaoxing’anling area in Northeast China. The *A. elata* roots were immediately placed in sterile plastic bags and stored at 4 °C. The endophytes were isolated within 48 h of collection. Before disinfection, the plant samples were thoroughly washed under running tap water for 10 h. The roots were surface-disinfected with 70% (*v*/*v*) ethanol for 0.5–1 min, 5% NaOCl for 5–10 min, 70% (*v*/*v*) ethanol for 0.5–1 min and burning for 10–30 s. The samples were subsequently rinsed with sterile water, and the outer tissue was removed with a sterile scalpel. Small pieces (0.5 × 0.5 cm) of *A. elata* were placed in Petri dishes containing malt extract agar (Oxoid-Unipath Ltd., Hampshire, UK), Czapeck agar (Oxoid-Unipath Ltd., Hampshire, UK), or potato dextrose agar (Oxoid-Unipath Ltd., Hampshire, UK), and were incubated at 28 °C for seven days. Following the incubation, single colonies of distinctive morphotypes were isolated on the basis of their morphological characteristics and appearance. The colonies were subsequently re-isolated by plating on PDA and incubating at 28 °C for 24–48 h to obtain pure cultures. All of the isolates were vacuum freeze-dried and deposited in the collection of the College of Life Sciences, Northeast Forestry University.

### 3.2. DNA Extraction and PCR Amplification of the 28S rRNA Gene

F ungal genomic DNA was extracted using the EZNA Fungal DNA Mini Kit (OMEGA, USA) according to the manufacturer’s instructions. The 50 μL PCR mixtures contained 15 ng of template DNA, 1× PCR buffer (Mg^2+^ free), 0.16 mM of each dNTP, 1.5 mM MgCl_2_, 0.45 μM of each primer, and 1 U of Takara rTaq DNA polymerase (Takara, Japan). The primers employed for the amplification of the D1/D2 region of the fungal 28S rRNA gene were NL1 (5′-GCATATCAATAAGCGGAGGAAAAG-3′) and NL4 (5′-GGTCCGTGTTTCAAGACGG-3′) [28]. The thermocycling program consisted of initial DNA denaturation at 95 °C for 5 min followed by 30 cycles of denaturation at 95 °C for 1 min, annealing at 52 °C for 45 s, and elongation at 72 °C for 1 min 30 s, ending with a final elongation step at 72 °C for 6 min [29].

The PCR amplification products were separated by electrophoresis through 1% (*w*/*v*) agarose gels, stained with ethidium bromide and visually examined under UV light. The PCR products were purified using the Agarose Gel DNA Extraction Kit (Takara, Japan) and sequenced by Sangon Biotech (Shanghai, China).

### 3.3. Phylogenetic Analysis and Nucleotide Sequence Accession Numbers

The sequences generated in this study were compared with those in GenBank [30]; those sequences with ≥99% similarity to the 28S rDNA D1/D2 regions (approximately 600 bp) were considered to belong to identical genera and were included in the alignment. A neighbor-joining tree was constructed using MEGA 5.0 software [31]. The number of bootstrap replications was 1000. The sequences were deposited in GenBank under the accession numbers listed in Table 1.

### 3.4. Determination of Triterpenoid Saponins

Each isolate was inoculated into 100 mL of PDA liquid medium (250 mL flask) and stirred at 150 rpm at 28 °C for two weeks. After ultrasonication, the supernatant was separated from the cell debris by centrifugation at 4000× *g* for 20 min. A 20 mL aliquot of the supernatant was poured into a 50 mL centrifuge tube (Corning Inc., Corning, NY, USA), and 20 mL of ethyl acetate was added to the same tube. After mixing, ultrasonication and incubation for 5 min, 5 mL of the supernatant was evaporated to dryness under a vacuum at 50 °C. The residue was dissolved in 2 mL of methanol. The methanol solutions were centrifuged at 4000× *g* for 10 min, and the supernatants were used for subsequent analysis of the total saponin content.

The measurement of the total extracted saponins was based on a color reaction of the acid-hydrolysis products of the saponins (*i.e.*, sapogenins) with vanillin. In total, 5 mL of the supernatant was added to a test tube and evaporated at 60 °C in a water bath. The residue was dissolved in 0.2 mL of 5% vanillin, mixed with 0.8 mL of perchloric acid, incubated in a 60 °C water bath for 15 min and quickly cooled in ice water. The concentration (mg/mL) of saponins in the reaction sample was determined using a spectrophotometer at 560 nm and comparing the readings against a calibration curve established with an oleanolic acid standard (National Institute for the Control of Pharmaceutical and Biological Products, Beijing, China) [32].

### 3.5. Antimicrobial Activity of the Representative Strains

The antimicrobial activity of typical strains after 14-day cultivation against 8 microorganisms (listed in Table 3) was assessed by the agar diffusion method [33]. Three 6 mm wells were made in each disk. With the exception of *Fusarium sporotrichioides* (isolated in our lab), the strains were purchased from the Agricultural Culture Collection of China (ACCC). Streptomycin sulfate (5 mg/well), amoxicillin (5 mg/well) and itraconazole (4.4 mg/well) were used as positive antimicrobial controls. The activity of the extracts was estimated from the diameter (mm) of the zone of inhibition.

### 3.6. Ginsenosides Analyses

One hundred milliliters of ethyl acetate was added to 100 mL liquid culture. Following 30 min of agitation at 160 rpm and ultrasonication at 50 °C, the supernatant was separated from the cell debris by centrifugation at 4000× *g* for 30 min. After evaporation, the pellet was dissolved in 5 mL of methanol, followed by filtration through a SepPak C-18 Cartridge (Waters, Milford, MA, USA). The following water/acetonitrile gradient system was employed during HPLC analysis: 0 min, 18% acetonitrile and 82% water; 40 min, 18% acetonitrile and 82% water; 50 min, 22% acetonitrile and 78% water; 70 min, 28% acetonitrile and 72% water; 100 min, 38% acetonitrile and 62% water; and 110 min, 18% acetonitrile and 82% water.

## 4. Conclusions

This study focused on the diversity of endophytic fungi from *A. elata* in Northeast China for the first time. Ninety-six strains were isolated. They were belonged to 12 genus respectively. The most abundant genera were *Diaporthe* and *Alteraria* represented by 25 and 12.5% of the isolates respectively. The analysis from saponins showed that many isolated fungi had the capacity to produce saponins. The highest concentration of saponins was found in G22 (*Penicillium* sp., 2.049 mg/mL), Ginsenoside Rb2 and Re were detected in the G22.This result indicate that G22 has the capacity to produce ginsenosides and consequently has application potentials.

## Figures and Tables

**Table 1:** Similarity between the isolates and closest species in GenBank.

Strain ID (Strains)	Accession no.	Closest species (Accession no.)	Coverage (%)
P17 (24)	JQ807963	Fungal sp. (GU552516)	100
		*Diaporthe* sp. (DQ377874)	100
		*Diaporthe eres* (AF362565)	100
G44-1 (12)	JQ807974	Uncultured *Alternaria* (JF495245)	100
		Uncultured *Alternaria* (JF449889)	100
		*Alternaria* sp. (GU048607)	100
G22 (8)	JQ807910	*Penicillium* sp. (GU048572)	100
		*Penicillium* sp. (DQ123664)	100
		*Penicillium rivolii* (GU033419)	99.3
G27 (8)	JQ807969	*Cladosporium* sp. (FJ790290)	100
		*Cladosporium cladosporioides* (AY213695)	100
		*Passalora fulva* (AB100653)	100
P6 (6)	JQ807984	*Schizophyllum commune* (AM269871)	99.8
		*Schizophyllum commune* (AB428351)	99.8
		*Schizophyllum commune* (AB363767)	99.8
P11(5)	JQ807967	Uncultured *Dictyochaeta* (JF449595)	99.3
		Uncultured *Dictyochaeta* (JF449592)	98.8
		*Dictyochaeta simplex* (AF178559)	98
P18(5)	JQ807982	Fungal sp. (GU552507)	100
		*Camarosporium leucadendri* (EU552106)	99.7
		*Camarosporium brabeji* (EU552105)	99.7
G3 (5)	JQ807935	*Nectria haematococca* (HM042416)	100
		*Nectria haematococca* (AB373719)	100
G41 (4)	JQ807972	*Cladosporium cladosporioides* (JN651416)	100
		*Cladosporium silenes* (JF770463)	100
		Uncultured *Cladosporium* (JF449832)	100
G46 (4)	JQ807956	*Fusarium avenaceum* (JN938913)	100
		*Fusarium* sp. (JF700486)	100
		*Fusarium avenaceum* (HM068325)	100
P37 (3)	JQ807942	*Peniophora* sp. (HM595610)	99.8
		*Peniophoraceae* sp. (AB576771)	99.8
		*Peniophoraceae* sp. (DQ094783)	99.8
P24(3)	JQ807922	Uncultured soil *fungus* (JQ311518)	99.5
		*Trichoderma parareesei* (JN874490)	99.3
		*Trichoderma* sp. (AB373724)	99.1
P16 (3)	JQ807976	Uncultured *Pleosporales* (JF691161)	100
		Uncultured *Epicoccum* (JF449817)	100
		Uncultured *Epicoccum* (JF449816)	100
P36 (2)	JQ807968	Uncultured *Cryptosporiopsis* (JF519417)	100
		Uncultured *Cryptosporiopsis* (JF519423)	99.8
		*Cryptosporiopsis ericae* (AY853167)	99.8
G5 (2)	JQ807983	Fungal sp. (GU552486)	100
		*Botryosphaeria dothidea* (AB454278)	100
		*Botryosphaeria dothidea* (FJ755233)	100
G49 (1)	JQ807918	Uncultured soil *fungus* (EU691410)	100
		Uncultured soil *fungus* (EU691436)	100
		Uncultured *fungus* (EF639724)	100
G39 (1)	JQ807981	*Corynespora smithii* (GU323201)	94.6
		*Fenestella fenestrate* (GU205220)	94.6
		*Phoma* sp. (JF746158)	94.3

**Table 2:** Triterpenoid saponin production of the representative isolate from each group.

Isolate ID	Mean ± Stdev (mg/mL) (*p* < 0.05)	Expected species
G22	2.049 ± 0.044 a	*Penicillium* sp.
P11	0.162 ± 0.004 b	*Dictyochaeta* sp.
P18	0.156 ± 0.006 b	*Camarosporium leucadendri*
G27	0.131 ± 0.003 c	*Cladosporium* sp.
P6	0.120 ± 0.003 cd	*Schizophyllum commune*
G49	0.113 ± 0.005 cd	Uncultured soil *fungus*
P37	0.109 ± 0.006 d	*Peniophora* sp.
P17	0.084 ± 0.005 e	*Diaporthe* sp.
P16	0.066 ± 0.005 ef	*Epicoccum* sp.
G41	0.066 ± 0.006 efg	*Cladosporium cladosporioides*
G46	0.065 ± 0.003 efg	*Fusarium avenaceum*
P24	0.060 ± 0.006 fg	*Trichoderma parareesei*
G44-1	0.059 ± 0.002 fg	*Alternaria* sp.
G39	0.048 ± 0.001 gh	*Corynespora smithii*
G5	0.038 ± 0.004 h	*Botryosphaeria dothidea*
G3	0.032 ± 0.005 h	*Nectria haematococca*
P36	0.030 ± 0.002 h	*Cryptosporiopsis* sp.

**Table 3:** Antimicrobial activity of representative endophytic fungi strains.

Test strains	Representative endophytic strains		

	G22	P11	P18
*Staphylococcus aureus* ACCC10499	++	+	−
*Bacillus subtitis* ACCC10243	−	−	−
*Klebsiella pneumoniae* ACCC10498	−	+++	+
*Pseudomonas aeruginosa* ACCC10500	−	−	−
*Phytophthora cactorum* ACCC36421	−	−	−
*Rhizoctonia solani* ACCC36233	−	+	−
*Aspergillus niger* ACCC30005	−	−	−
*Fusarium sporotrichioides*	−	+	−

(−) no inhibition; (+) presence of a zone of growth inhibition +++ width of growth inhibition zone > 10 mm, ++ 5–10 mm, + 1–5 mm.
